# Differences in Radiological and Pathological Findings by ANCA‐Subtype in ANCA‐Positive Idiopathic Interstitial Pneumonias

**DOI:** 10.1111/crj.70061

**Published:** 2025-03-12

**Authors:** Tetsuro Sawata, Susumu Sakamoto, Yusuke Usui, Aika Suzuki, Hideya Kitamura, Tae Iwasawa, Shoichiro Matsushita, Yasuhiro Terasaki, Shinobu Kunugi, Kazuma Kishi, Tomoyuki Fujisawa, Takafumi Suda, Sakae Homma

**Affiliations:** ^1^ Department of Respiratory Medicine AOI Universal Hospital Kawasaki Japan; ^2^ Department of Respiratory Medicine Toho University Graduate School of Medicine Ōta Japan; ^3^ Department of Respiratory Medicine Kanagawa Cardiovascular and Respiratory Center Yokohama Japan; ^4^ Department of Radiology Kanagawa Cardiovascular and Respiratory Center Yokohama Japan; ^5^ Department of Radiology St. Marianna University School of Medicine Kawasaki Japan; ^6^ Division of Pathology Nippon Medical School Hospital Tokyo Japan; ^7^ Department of Analytic Human Pathology Nippon Medical School Tokyo Japan; ^8^ Second Division, Department of Internal Medicine Hamamatsu University School of Medicine Hamamatsu Japan; ^9^ Department of Advanced and Integrated Interstitial Lung Diseases Research, School of Medicine Toho University Ōta Japan

**Keywords:** anti‐neutrophil cytoplasmic antibody (ANCA), idiopathic interstitial pneumonias, myeloperoxidase (MPO)‐ANCA, proteinase 3 (PR3)‐ANCA

## Abstract

**Introduction:**

Anti‐neutrophil cytoplasmic antibody (ANCA) seropositivity strongly correlates to ANCA‐associated vasculitis. Patients with idiopathic interstitial pneumonias (IIPs) without systemic vasculitis are sometimes ANCA‐positive. Radiological and pathological differences between patients with myeloperoxidase (MPO)‐ANCA‐positive and those with proteinase 3 (PR3)‐ANCA‐positive IIPs remain unclear. To determine whether high‐resolution computed tomography (HRCT) features and pathology findings differ by ANCA subtype in ANCA‐positive IIP patients in a national database. Clinical, radiological, and pathological data were examined along with a web‐based multidisciplinary discussion.

**Methods:**

We reviewed records of 10 MPO‐ANCA‐positive and 9 PR3‐ANCA‐positive IIP patients who underwent HRCT and surgical lung biopsy between April 2009 and March 2014. Pulmonologists, chest radiologists, and pathologists evaluated HRCT scans and pathological findings independently. Patterns were classified using ATS/ERS/JRS/ALAT 2011 guidelines for idiopathic pulmonary fibrosis.

**Results:**

HRCT patterns were definite usual interstitial pneumonia (UIP) (*n* = 8; 42.1%), possible UIP (*n* = 6; 31.6%), and inconsistent with UIP (*n* = 5; 26.3%). Pathological patterns were definite UIP (n = 5; 26.3%), probable UIP (n = 8; 42.1%), possible UIP (*n* = 4; 21.1%), and not UIP (*n* = 2; 10.5%). HRCT and pathological patterns did not differ between MPO‐ANCA‐positive and PR3‐ANCA‐positive IIPs. Radiological features were reticulation (*n* = 13; 68.4%), nodules (*n* = 12; 63.1%), honeycombing (*n* = 10; 52.6%), and increased attenuation around honeycombing (*n* = 7; 36.8%). Pathological findings were cysts (*n* = 12; 63.1%), lymphoid follicles with germinal centers (*n* = 11; 57.9%), and peribronchiolar wall lymphocytic infiltration (*n* = 11; 57.9%).

**Conclusion:**

HRCT and pathological patterns did not differ between MPO‐ANCA‐positive and PR3‐ANCA‐positive IIPs. This absence of significant differences suggests a similar mechanism underlying both types of interstitial pneumonia.

## Graphical abstract

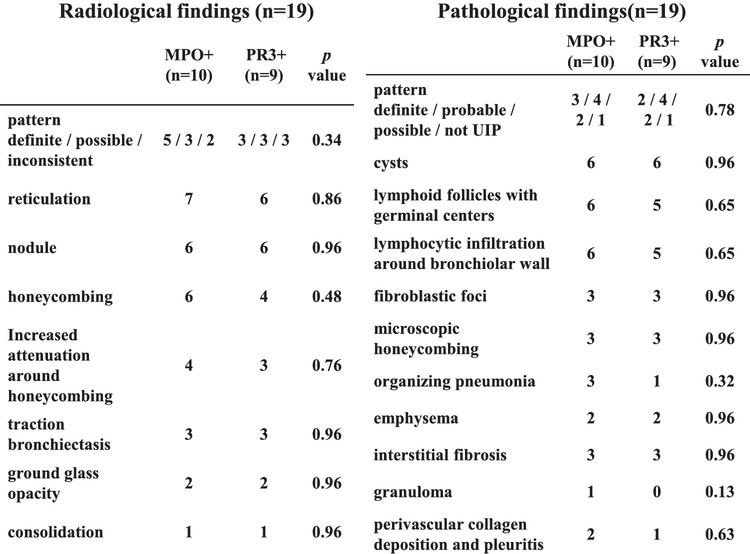

## 1 Introduction

ANCA‐associated vasculitis (AAV) describes a group of systemic diseases in which a necrotizing vasculitis of the small blood vessels has the potential to attack the upper and lower respiratory tracts, the skin, eyes, and kidneys [1]. The presence of anti‐neutrophil cytoplasmic antibodies (ANCAs) is strongly associated with AAV. ANCAs are autoantibodies directed against enzymes in the granules of polymorphonuclear leukocytes [1, 2]. Myeloperoxidase‐antineutrophil cytoplasmic antibody (MPO‐ANCA) and proteinase‐3‐antineutrophil cytoplasmic antibody (PR3‐ANCA) are autoantibodies against MPO and PR3, respectively, in the cytoplasm of neutrophils. Lung involvement has been reported in 25%–80% of patients with AAV [2], and the findings are diverse, including nodules, bronchiectasis, pleural effusion, emphysema, ground glass opacity, cavity, and interstitial pneumonia. However, ANCA seropositivity is sometimes seen in patients with idiopathic interstitial pneumonias (IIPs) without systemic vasculitis [3, 4, 5, 6, 7, 8, 9]. In Japanese patients with MPO‐ANCA‐associated vasculitis (JMAAV), a refractory vasculitis study group determined the efficacy of a severity‐based treatment protocol for MPO‐ANCA‐positive AAV (MPO‐AAV) [10]. Pulmonary‐limited AAV was defined as AAV without organ involvement outside of pulmonary lesions; based on this definition, 6 of 48 patients with MPO‐AAV (12.5%) had pulmonary‐limited AAV. However, there is currently no consensus among pulmonologists as to whether MPO‐ANCA‐positive interstitial pneumonia without generalized organ involvement other than interstitial pneumonia should be included in the diagnosis of pulmonary‐limited AAV. Accordingly, MPO‐ANCA‐positive interstitial pneumonia may include not only microscopic polyangiitis and unclassified AAV [11] but also MPO‐ANCA‐positive interstitial pneumonia without systemic vasculitis manifestations, as well as pulmonary‐limited AAV preceding the onset of AAV. Some radiological and pathological studies have been conducted in patients with MPO‐ANCA‐positive IIPs, and radiological studies have been conducted on those with PR3‐ANCA‐positive IIPs. However, there have been no pathological studies in patients with PR3‐ANCA‐positive IIPs. Also, the differences in radiological features and pathological findings between patients with MPO‐ANCA‐positive IIPs and those that are PR3‐ANCA‐positive remain unclear. We recently developed a nationwide cloud‐based database with clinical, radiological, and pathological data of patients with biopsy‐proven IIP and a web‐based multidisciplinary discussion (MDD) system [12]. This facilitates establishing an accurate diagnosis of IIP. Thus, this study aimed to clarify whether high‐resolution computed tomography (HRCT) and pathological findings vary by ANCA subtype in ANCA‐positive patients with a diagnosis of IIP enrolled in a nationwide cloud‐based integrated database with clinical, radiological, and pathological data. The study also included a web‐based MDD system.

## 2 Materials and Methods

### 2.1 Subjects

**FIGURE 1:**
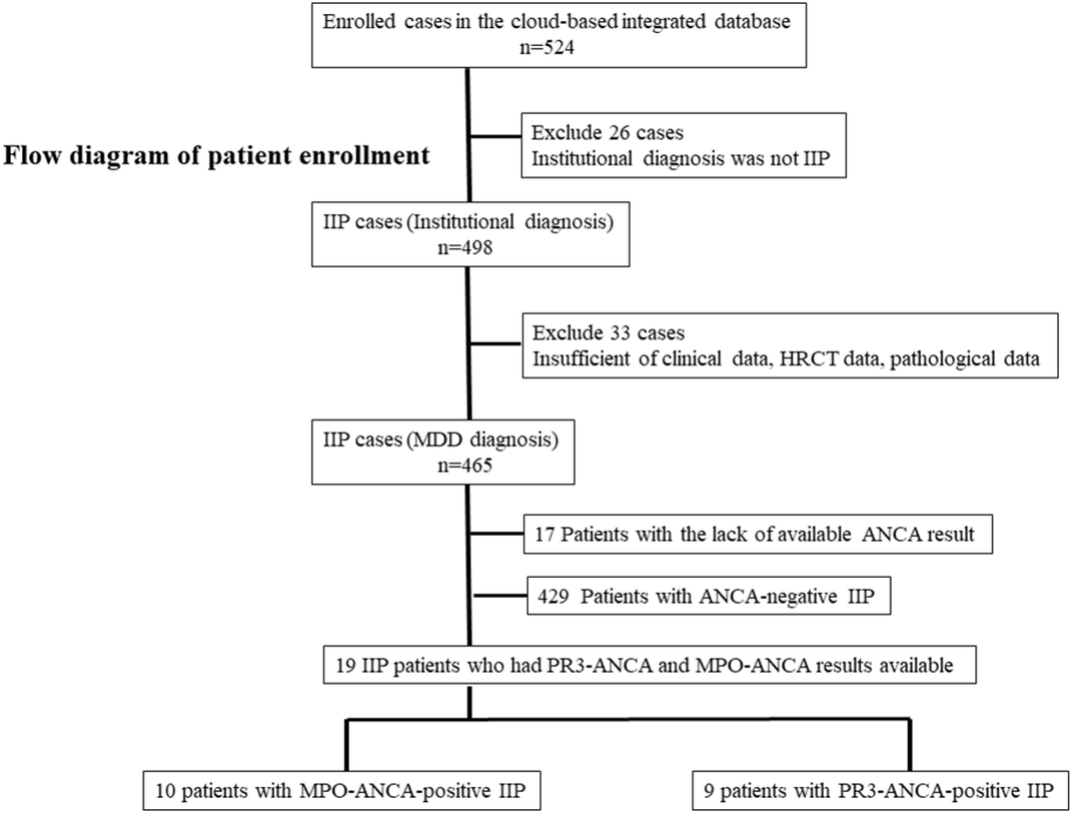
Flow diagram of patient enrollment.

The study assessed records of patients diagnosed with IIP at their respective institutions and who had undergone HRCT of the chest and surgical lung biopsy (SLB) between April 2009 and March 2014. Details of the methods used to develop the database have been described previously [12]. Briefly, this is a secondary analysis of data from a retrospective cohort study for which we developed a nationwide cloud‐based integrated database containing clinical, radiological, and pathological data of IIP patients according to a web‐based MDD diagnosis [12]. The Japanese Respiratory Society selected a total of 39 participating institutions for this study. A flow diagram of enrollment and grouping of our study patients is shown in Figure 1. Of the 524 cases of IIP registered in the Hamamatsu University School of Medicine cloud‐based integrated database, 26 were excluded because the institutional diagnosis was not IIP. Of the remaining 498 cases, 33 were excluded because of missing or incomplete clinical, HRCT, and/or pathological data. Of the resulting 465 cases, 17 were further excluded because of unavailable ANCA results; 429 patients with ANCA‐negative IIP were excluded. Finally, the study included 10 patients with MPO‐ANCA‐positive interstitial pneumonia and 9 patients with PR3‐ANCA‐positive interstitial pneumonia for whom searchable clinical, radiological, and pathological information were available. Patients in these two groups were compared in terms of clinical, radiological, and pathological findings. Medical records were assessed to obtain clinical data, which included patient characteristics, laboratory data, and pulmonary function at the time of diagnosis.

The institutional review board of Toho University Graduate School of Medicine approved this study (Approval Number: M19146). The need for patient assent or informed consent was waived because the study involved a retrospective review of clinical records.

Diagnoses of IIPs, including idiopathic pulmonary fibrosis (IPF), idiopathic nonspecific interstitial pneumonia (iNSIP), cryptogenic organizing pneumonia, unclassifiable IIP, and other IIPs, were based on clinical history, physical examination, and HRCT findings, with or without histologic examination, in accordance with international consensus criteria [13, 14, 15].

#### 2.1.1 Review of Radiological Findings

All HRCT data were obtained within 3 months before SLB, and the procedure was carried out for each patient during single breath‐holding at full inspiration; 1‐ to 5‐mm‐thick samples at 1‐ to 5‐mm slice intervals were collected throughout the lungs. Initially, all images were collected as digital imaging and communications in medicine (DICOM) files. Next, DICOM data were converted into a compact disc read‐only memory format for input as electronic medical records at our hospital. All images were analyzed using a 3D image analysis system SYNAPSE VINCENT version 3 (Fujifilm Medical Systems, Tokyo, Japan). HRCT patterns were classified according to the 2011 IPF guidelines as usual interstitial pneumonia (UIP), possible UIP, and inconsistent with UIP [13]. HRCT scans were analyzed for the following features: reticulation, traction bronchiectasis, honeycombing, ground glass opacity, consolidation, increased attenuation around honeycombing, and nodules. These features were selected with reference to previous studies [9, 16, 17, 18, 19].

#### 2.1.2 Review of Pathological Findings

Pathological sections of SLB specimens were stained with hematoxylin and eosin and elastic van Gieson stains. The pathological classifications of UIP, namely, probable UIP, possible UIP, and not UIP, were based on the IPF guidelines [13]. The pathological features evaluated were: cyst, lymphoid follicles with germinal centers, lymphocytic infiltration around the bronchiolar wall, fibroblastic foci, microscopic honeycombing, organizing pneumonia, emphysema, interstitial fibrosis, perivascular collagen deposition, and pleuritis.

#### 2.1.3 Statistical Analysis

Results are presented as the mean ± standard deviation. The between‐group frequency was compared using either the Mann–Whitney *U* test or the chi‐squared test. A *p*‐value < 0.05 was regarded as statistically significant for all tests. All statistical analyses were performed using SPSS statistical software package (IBM SPSS Statistics version 19; IBM Corp, Armonk, NY).

## 3 Results

### 3.1 Baseline Characteristics

Among the IIP patients evaluated in this study, MPO‐ANCA‐positive IIP was observed in 10 cases (2.2%) and PR3‐ANCA‐positive IIP in 9 cases (2.0%). Clinical characteristics of MPO‐ANCA‐positive IIP and PR3‐ANCA‐positive IIP patients are summarized in Table 1. There were no statistically significant differences in gender, age at IIP diagnosis, smoking history, IIP diagnosis, laboratory data, PaO_2_, and pulmonary function test results between the two groups.

**TABLE 1:** Clinical characteristics in patients with MPO‐ANCA‐positive and PR3‐ANCA‐positive‐interstitial pneumonia.

	MPO+ (*n* = 10)	PR3+ (*n* = 9)	*p*
Gender (male/female)	8/2	5/4	0.98
Age	57.6 ± 5.9 (38–75)	67.0 ± 7.4 (58–76)	0.11
Smoking (never/current, ex)	4/6	4/5	0.81
Pack‐years	32.5 ± 5.3	24.7 ± 4.6	0.64
IIPs diagnosis (IPF/NSIP/unclassifiable)	3/4/3	2/2/5	0.16
KL‐6 (U/mL)	1133 ± 457	1484 ± 411	0.84
SP‐D (ng/mL)	175 ± 108	219 ± 96	0.65
LDH (IU/mL)	224 ± 89	244 ± 75	0.41
PaO2 (Torr) (RA)	86.2 ± 12.2	90.7 ± 11.6	0.65
%FVC (%)	81.6 ± 17.0	83.2 ± 15.8	0.54
FEV1% (%)	75.5 ± 10.5	93.1 ± 15.8	0.23
%DLco (%)	68.0 ± 15.1	64.2 ± 16.1	0.75

*Note:* Data are presented as mean ± standard deviation.

Abbreviations: ANCA, anti‐neutrophil cytoplasmic antibody; DLco, diffusing capacity of the lungs for carbon monoxide; FEV1, forced expiatory volume in 1 s; FVC, forced vital capacity; IPF, idiopathic pulmonary fibrosis; KL‐6, Krebs von den Lugen‐6; LDH, lactate dehydrogenase; MPO, myeloperoxidase; NSIP, nonspecific interstitial pneumonia; PaO_2_, partial pressure of arterial oxygen; PR‐3, proteinase 3; RA, room air; SP‐D, surfactant protein‐D.

### 3.2 Radiological and Pathological Findings

**FIGURE 2:**
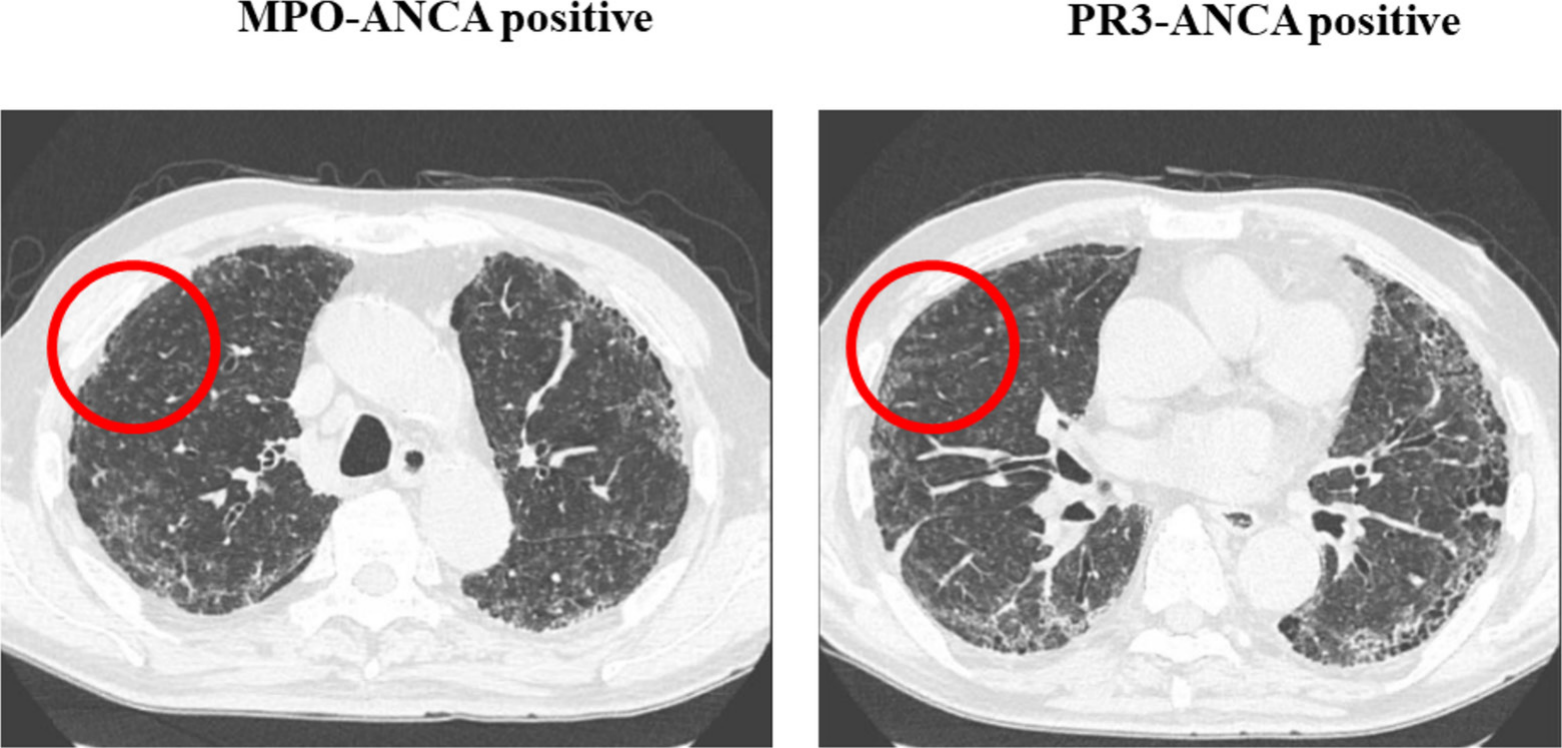
Radiological findings of nodular lesions.

**FIGURE 3:**
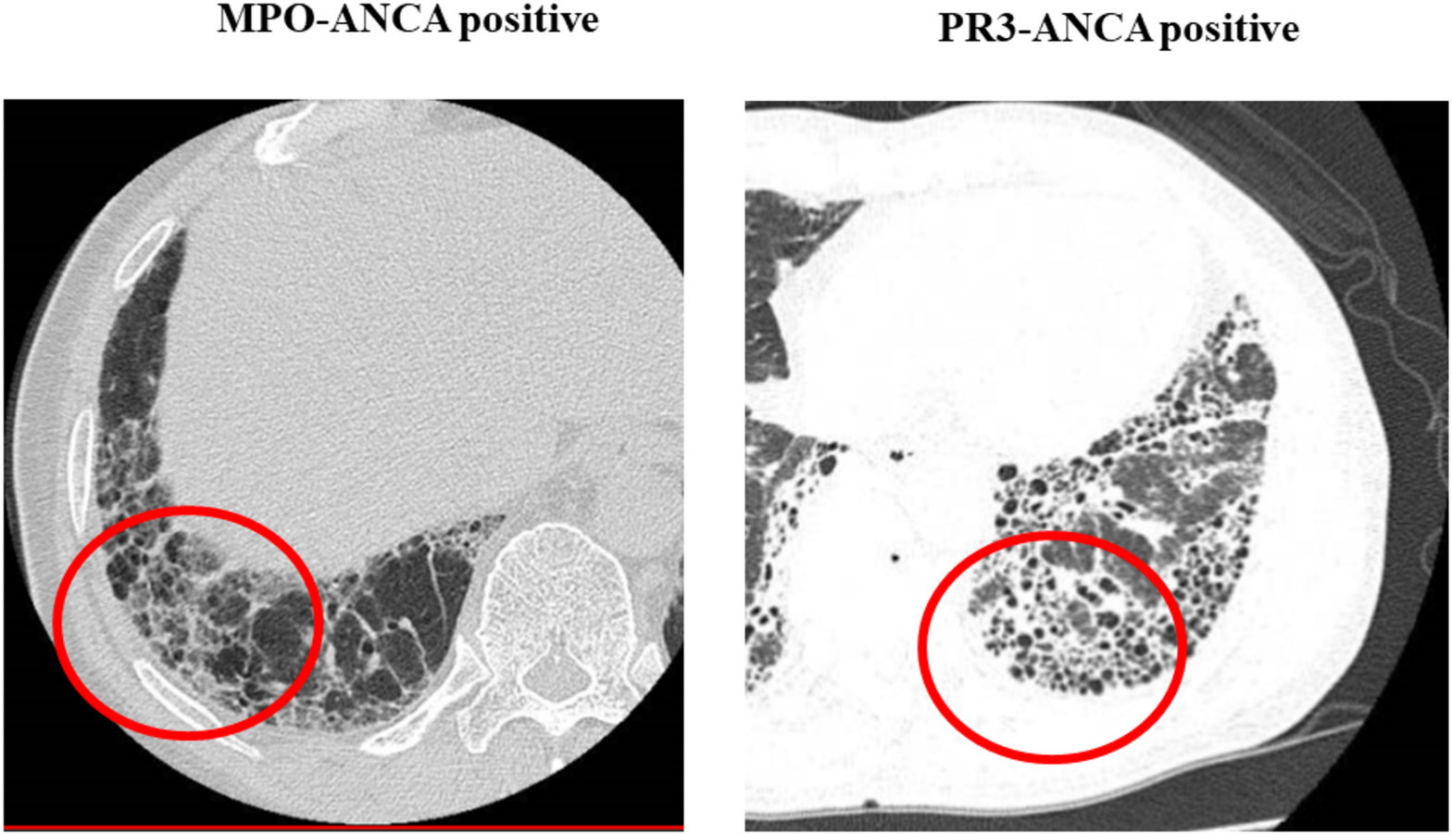
Radiological findings of increased attenuation around honeycombing.

**FIGURE 4:**
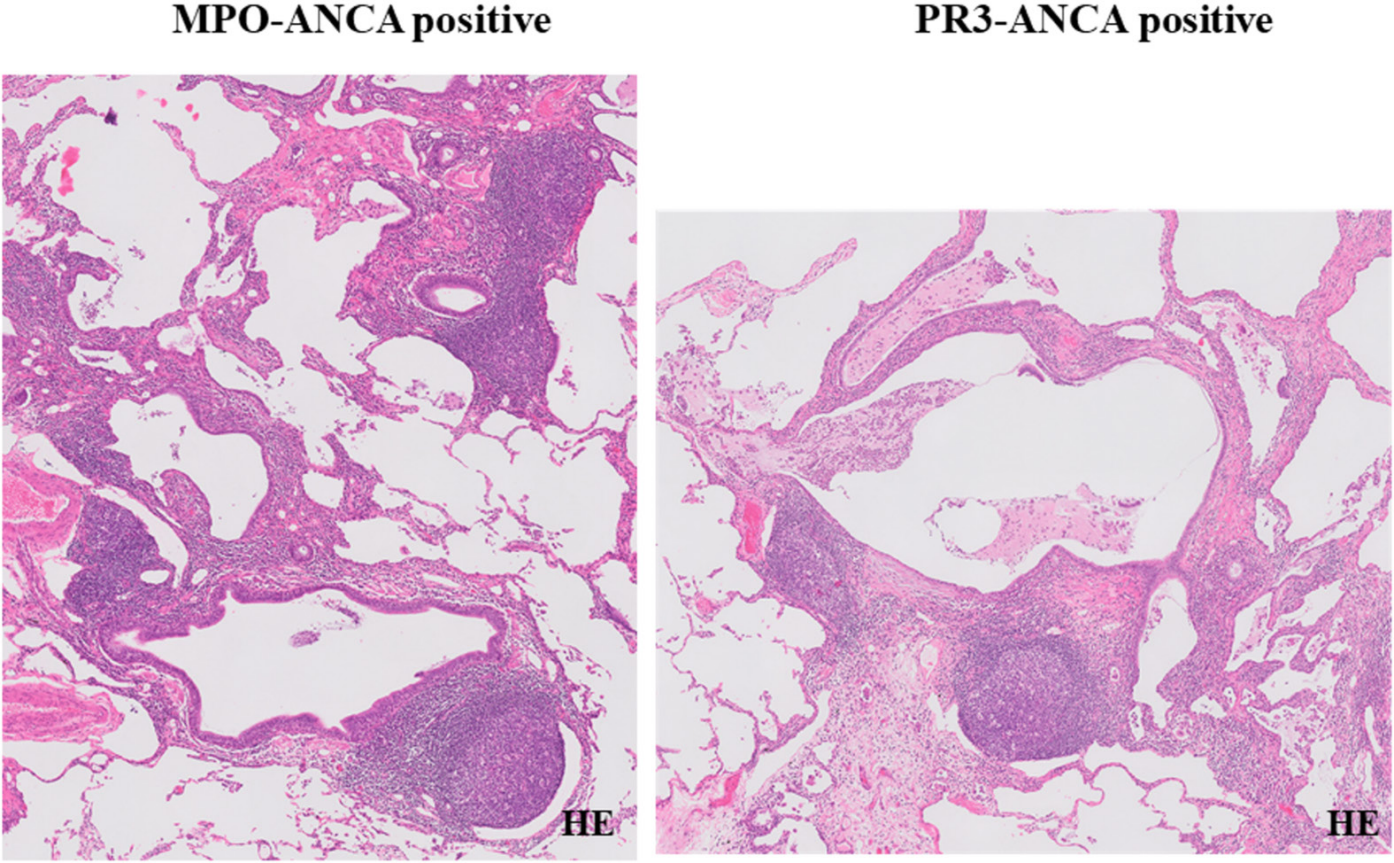
Pathological findings of nodular lesions on HRCT image showing lymphocytic infiltration and lymphoid follicles with germinal center around bronchiolar walls.

**FIGURE 5:**
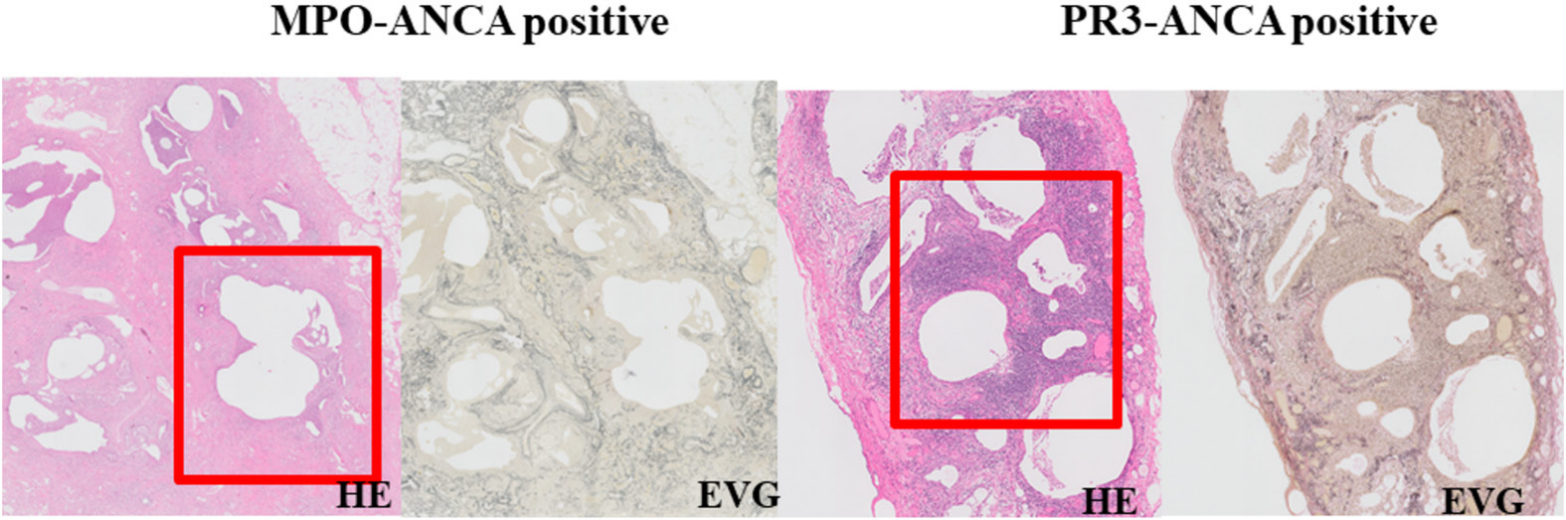
Pathological findings of increased attenuation around honeycombing on HRCT image showing lymphocytic infiltration of the bronchiolar walls inducing destructive bronchiolitis resulting cyst formation.

HRCT and pathological patterns and the presence/absence of specific radiological features and pathological findings are described in Tables 2 and 3, respectively. HRCT patterns were definite UIP (*n* = 8; 42.1%), possible UIP (*n* = 6; 31.6%), and inconsistent with UIP (*n* = 5; 26.3%). The main radiological findings were reticulation (*n* = 13; 68.4%), nodular lesion (*n* = 12; 63.1%), honeycombing (*n* = 10; 52.6%), and increased attenuation around honeycombing (*n* = 7; 36.8%). Pathological patterns were definite UIP (n = 5; 26.3%), probable UIP (n = 8; 42.1%), possible UIP (*n* = 4; 21.1%), and not UIP (*n* = 2; 10.5%) patients. The pathological patterns of the cases that were pathologically not UIP were fibrotic NSIP. The main pathological findings were cyst formation (*n* = 12; 63.1%), lymphoid follicles with germinal centers (*n* = 11; 57.9%), and lymphocytic infiltration around the bronchiolar wall (*n* = 11; 57.9%). Figure 2 shows nodular lesions, one of the main radiological findings in patients with MPO‐ANCA‐positive or PR3‐ANCA‐positive IIP. Figure 3 shows radiological findings of increased attenuation around honeycombing. Pathological findings of nodular lesions on HRCT are shown in Figure 4 while Figure 5 shows pathological findings of increased attenuation around honeycombing on HRCT. Bronchiolitis induced by lymphocytic infiltration of the bronchiolar walls, which triggers bronchiolar wall destruction, resulting in cyst formation. Patients considered as having honeycombing on HRCT were histologically found to have cyst formation with bronchiolitis and attendant destruction of bronchiolo‐alveolar structures; this is not observed in IPF/UIP.

**TABLE 2:** Radiological findings of MPO‐ANCA‐ and PR‐3ANCA‐positive patients (*n* = 19).

	MPO+ (*n* = 10)	PR3+ (*n* = 9)	*p*
Pattern (definite/possible/inconsistent)	5/3/2	3/3/3	0.34
Reticulation	7	6	0.86
Nodule	6	6	0.96
Honeycombing	6	4	0.48
Increased attenuation around honeycombing	4	3	0.76
Traction bronchiectasis	3	3	0.96
Ground glass opacity	2	2	0.96
Consolidation	1	1	0.96

Abbreviations: MPO, myeloperoxidase; PR‐3, proteinase 3.

**TABLE 3:** Pathological findings of MPO‐ANCA‐ and PR‐3ANCA‐positive patients (*n* = 19).

	MPO+ (*n* = 10)	PR3+ (*n* = 9)	*p*
Pattern (definite/probable/possible/not UIP)	3/4/2/1	2/4/2/1	0.78
Cysts	6	6	0.96
Lymphoid follicles with germinal centers	6	5	0.65
Lymphocytic infiltration around bronchiolar wall	6	5	0.65
Fibroblastic foci	3	3	0.96
Microscopic honeycombing	3	3	0.96
Organizing pneumonia	3	1	0.32
Emphysema	2	2	0.96
Interstitial fibrosis	3	3	0.96
Perivascular collagen deposition and pleuritis	2	1	0.63

Abbreviations: MPO, myeloperoxidase; PR‐3, proteinase 3; UIP, usual interstitial pneumonia.

### 3.3 Comparison Between MPO‐ANCA‐Positive and PR3‐ANCA‐Positive Patients

There was no difference in HRCT and pathological patterns between MPO‐ANCA‐positive and PR3‐ANCA‐positive patients.

## 4 Discussion

This study compared clinical, imaging, and pathological findings among patients with ANCA‐positive interstitial pneumonia. In this study, we found that the HRCT patterns were definite UIP (*n* = 8; 42.1%), possible UIP (*n* = 6; 31.6%), and inconsistent with UIP (*n* = 5; 26.3%). The main findings were reticulation, nodular lesion, honeycombing, and increased attenuation around honeycombing. In 9 MPO‐ANCA‐positive patients among 61 patients with IPF (14.8%), the main HRCT findings were reticular opacities, traction bronchiectasis, and honeycombing, all of which were also observed in MPO‐ANCA‐negative patients [7]. In another study of 31 patients with MPO‐ANCA‐positive interstitial pneumonia, 26 patients demonstrated honeycombing on HRCT consisting primarily of lower lung field‐dominant reticulation from directly below the pleura to the outer layer of the lung [6]. Furthermore, an examination of 12 patients with MPO‐ANCA‐positive UIP revealed cysts and increased attenuation around honeycombing in comparison with patients with IPF [9]. A report noted that in 16 patients with PR3‐ANCA‐positive UIP, HRCT patterns reflected UIP in 18.8% of patients, possible UIP in 18.8% of patients, NSIP in 31.3% of patients, and were unclassifiable in 31.3% of patients [20]. In this study, pathological patterns were definite UIP (*n* = 5; 26.3%), probable UIP (*n* = 8; 42.1%), possible UIP (*n* = 4; 21.1%), and not UIP (*n* = 2; 10.5%). Several previous studies examined the histopathology of MPO‐ANCA‐positive interstitial pneumonia. In an examination of 13 patients with MPO‐ANCA‐positive interstitial pneumonia, Homma et al. reported that all patients demonstrated a UIP pattern, with 23% of those also demonstrating mixed cellular NSIP [6]. In another study, while UIP was a common histological pattern in interstitial pneumonia, NSIP was also observed. However, in comparison with the pattern of IPF/UIP, differences in histopathological images were evident; in MPO‐ANCA/UIP, the interstitium demonstrates mononuclear cell infiltration, particularly an increase in plasma cells; hyperplasia of lymphoid follicles with germinal centers, cellular bronchiolitis, and cyst formation are also prominent [9]. Similarly, in our study, the main findings were lymphocytic infiltration around the bronchiolar wall, lymphoid follicles with germinal centers, and cyst formation. Images demonstrated centrilobular nodules that reflected lymphocytic infiltration around the bronchioles and lymphoid follicles with germinal centers. In MPO‐ANCA‐positive interstitial pneumonia, the presence of bronchiolar lesions is frequently reported from HRCT images, and histopathological findings suggestive of bronchiolitis are frequently seen [8, 9]. Takemura et al. reported destruction of the structure of the bronchiolar wall of membranous bronchioles by marked mononuclear cell infiltration. This resembles the cellular/destructive bronchiolitis observed with pulmonary nodules in rheumatoid arthritis; due to inflammatory cell infiltration, there is loss of the bronchiolar wall structure, with resulting destruction of acinar and lobular structures [21, 22, 23]. In this study, we observed similar findings in PR3‐ANCA‐positive patients. Regarding pathological findings of cyst formation, honeycombing was observed on HRCT. Pronounced cyst formation is frequently observed in MPO‐ANCA‐positive interstitial pneumonia [9, 21]; however, the origin and progression of these cystic lesions have not been sufficiently investigated. Based on pathological examination, Takemura et al. considered the above‐described bronchiolitis and attendant destruction of bronchiolo‐alveolar structures to be crucial for cyst formation and examined those pathological images [21, 22]. Their examination considered the infiltration of inflammatory cells into the lobular bronchioles (below the terminal bronchioles) along with the resulting destruction of bronchiolar wall structure and alveolar destruction to be crucial for cyst formation. In our study, cases considered to have honeycombing on HRCT were histologically found to have cyst formation with bronchiolitis and attendant destruction of alveolar structures, which is not observed in IPF/UIP. The honeycombing of IPF/UIP is accompanied by smooth muscle growth in the fibrotic areas and aggregation and fusion loss of alveolar wall elastic fibers. Finally, dense fibrosis causes the alveolar ducts in the center of the lobes and lobules to dilate onto the alveoli, forming alveoli of the same diameter. On the other hand, with regard to the cysts in ANCA‐positive interstitial pneumonia examined in this study, they were thought to form as a result of bronchiolitis causing destruction of the bronchial wall. Thus, it is suggested that the mechanism of the honeycombing in IPF/UIP is different from that of the cysts in ANCA‐positive IP. Going forward, detailed research will be necessary regarding changes in imaging features from bronchiolitis to cyst formation and corresponding pathological images. To our knowledge, our study is the first to examine pathological images in PR3‐ANCA‐positive interstitial pneumonia and to compare MPO‐ANCA‐positive and PR3‐ANCA‐positive patients; no study has yet done so to date. The absence of significant differences between MPO‐ANCA‐positive and PR3‐ANCA‐positive cases suggests that the same mechanism underlies both types of interstitial pneumonia. Going forward, it will be necessary to make further comparison among more cases of MPO‐ANCA‐positive and PR3‐ANCA‐positive interstitial pneumonia.

This study has several limitations. First, only a small number of patients were examined. Thus, our findings may not be extrapolated to a larger population. Nevertheless, this is the first study to conduct a histological examination in PR3‐ANCA‐positive interstitial pneumonia in comparison with MPO‐ANCA‐positive pneumonia. Second, interstitial pneumonia was assessed only at diagnosis, and disease progression was not tracked. Future studies will thus require comparisons among patients who develop MPA with those who do not develop MPA after a diagnosis of interstitial pneumonia. Furthermore, such studies should also examine treatment responsiveness and disease prognosis.

In conclusion, the main radiological findings were reticulation, nodular lesions, honeycombing, and increased attenuation around honeycombing. The main pathological findings were cyst formation, lymphoid follicles with germinal centers, and lymphocytic infiltration around the bronchiolar wall. HRCT and pathological patterns did not differ between MPO‐ANCA‐positive and PR3‐ANCA‐positive IIPs. This absence of significant differences suggests that the same mechanism underlies changes from bronchiolitis to cyst formation in both types of interstitial pneumonia.

## Author Contributions

T. Sawata designed the study; T. Sawata, S. Sakamoto, and S. Homma performed the experiments; S. Sakamoto and S. Homma contributed important reagents; T. Sawata and T. Suda collected data; T. Sawata, S. Sakamoto, Y. Usui, A Suzuki, H. Kitamura, T. Iwasawa, S. Matsushita, Y. Terasaki, S. Kunugi, K. Kishi, T. Fujisawa, T. Suda and S. Homma analyzed the data; T. Sawata wrote the paper; and S. Sakamoto, S. Homma, and K. Kishi reviewed the manuscript.

## Conflicts of Interest

The authors declare no conflicts of interest.

## Data Availability

All data generated or analyzed during this study are included in this article.
